# American Sign Language Alphabet Recognition Using a Neuromorphic Sensor and an Artificial Neural Network

**DOI:** 10.3390/s17102176

**Published:** 2017-09-22

**Authors:** Miguel Rivera-Acosta, Susana Ortega-Cisneros, Jorge Rivera, Federico Sandoval-Ibarra

**Affiliations:** 1 Advanced Studies and Research Center (CINVESTAV), National Polytechnic Institute (IPN), Zapopan 45019, Mexico; marivera@gdl.cinvestav.mx (M.R.-A.); sandoval@gdl.cinvestav.mx (F.S.-I.); 2 CONACYT-Advanced Studies and Research Center (CINVESTAV), National Polytechnic Institute (IPN), Zapopan 45019, Mexico; jriverado@conacyt.mx

**Keywords:** feature extraction, contour detection, hand posture recognition

## Abstract

This paper reports the design and analysis of an American Sign Language (ASL) alphabet translation system implemented in hardware using a Field-Programmable Gate Array. The system process consists of three stages, the first being the communication with the neuromorphic camera (also called Dynamic Vision Sensor, DVS) sensor using the Universal Serial Bus protocol. The feature extraction of the events generated by the DVS is the second part of the process, consisting of a presentation of the digital image processing algorithms developed in software, which aim to reduce redundant information and prepare the data for the third stage. The last stage of the system process is the classification of the ASL alphabet, achieved with a single artificial neural network implemented in digital hardware for higher speed. The overall result is the development of a classification system using the ASL signs contour, fully implemented in a reconfigurable device. The experimental results consist of a comparative analysis of the recognition rate among the alphabet signs using the neuromorphic camera in order to prove the proper operation of the digital image processing algorithms. In the experiments performed with 720 samples of 24 signs, a recognition accuracy of 79.58% was obtained.

## 1. Introduction

Deafness is a very important problem that affects people both economically and socially because all communication between individuals is through languages. Its main objective is to share information between a transmitter and a receiver using various channels (speech, writing, or gestures). Most of the population uses speech communication; thus, the integration of deaf people into society is very difficult because of the general lack of knowledge of sign languages. According to the World Health Organization, there are 360 million people worldwide with hearing loss, of which 32 million are children [1], which represents approximately 5% of the population. Therefore, it is a global problem. Research into sign language translation could solve many of the problems that deaf people face every day, allowing a flow of communication between any person and those with hearing disabilities.

For the hand postures recognition problem, the hand contour is often used because it simplifies real-time performance. All of the current related work can be globally sorted into software and hardware based solutions. Several works can be found for the software based platforms, such as [2], which uses contours to obtain characteristic points to reduce the computation time, which is a similar approach to the system presented in this work; however, their interesting points extraction algorithms require many steps, such distance calculation between all of the pixels with the object contour, and some other full-frame scanning operations. Ghassem et al. [3] proposed a method to extract global hand features using the external shape, obtaining contour points between two consecutive convex hull vertices. However, their feature extraction method is not robust. In [4], an algorithm is used to represent objects in images with a fixed number of sample points, but they use 100 sample points to generate the descriptor of every contour, making their classification step very computationally complex. Lahamy and Lichti [5] presented a real-time hand posture recognition using a range camera that gives visual and distance data, using the closest pixels to the camera for hand area segmentation and a voxel representation on every incoming image to compare it with a database for classification. They reported a recognition rate of 93.88%; however, they used only 12 postures, avoiding consideration of the most similar American Sign Language (ASL) signs, e.g., “A”, “B”, “E”, among others. In addition, it was implemented on a desktop computer with software (sequential nature) and their hand descriptors contain a lot of data, which makes it computationally inefficient. Some researchers use more than one digital camera to obtain a 3D perception [6]. Although it has an accuracy of 95%, the high computer requirements and the marked gloves make it uncomfortable for real applications.

Recently, the Microsoft Kinect^®^ has been widely used [7,8,9] because of its ability to obtain depth information, which is very valuable for cropping the hand areas, if they overlap the face, allowing real-time applications, but a 53% of accuracy in ASL alphabet classification is reported, which is low nowadays. Additionally, the Kinect device requires a personal computer with the proper drivers installed. Cao Dong et al. [10] proposed a system that uses depth images with a Kinect device to create a hand skeleton for ASL alphabet recognition using a Random Forest learning model, reaching an accuracy near 90%. However, they require a colored glove to generate the training dataset. In [11], an FSL (French Sign Language) translation system is implemented as a web platform, whose application is wide because of the current availability of the internet, but their system uses a sign search engine based on an encoding of the FSL signs and is not based on image processing. Besides the power consumption and the many computational resources required, these programmed systems have a sequential nature; accordingly, the system could present a higher latency, making efficient real-time applications difficult. These research projects represent a huge advance in hand posture recognition tasks, but they are not intended to be applied in embedded platforms as most of them require a full computer, special colored gloves, and more than one sensor. Increasing the parallelism in the algorithms decreases the execution time. A common method for parallelizing the algorithms in software is to implement them in a Graphics Processing Unit (GPU) [12,13], although managing a GPU requires many computer resources and it cannot be used without an operating system with the proper drivers installed.

For the hardware based solution, the algorithm is custom designed in hardware such as the Field-Programmable Gate Array (FPGA) used in this work, offering the possibility of parallelizing the algorithm and improving the execution time without dramatically increasing the resources needed [14]. This device allows for designing and developing complex systems with high parallelization and pipelining levels, while simultaneously implementing a microprocessor that executes software, all in a single chip. Hence, the hardware-based solution is more suitable for improving algorithm execution and gaining a significant reduction in computational complexity. In [15,16], systems are proposed to recognize 24 ASL alphabet signs implemented in a FPGA using frame-based Red-Green-Blue (RGB) cameras; nevertheless, the need of a colored glove makes it uncomfortable for practical applications and the usage of periodical cameras means that these systems require more operations for a single frame, including the need for full-frame scanning operations. Wang et al. [17] propose different models of neural networks using stochastic logic for the implementation of human postures classification systems in FPGA.

There is another option in camera sensors that can be used for ASL recognition—the neuromorphic camera, which has an event-based behavior that only upgrades the pixel value when there is a light change in the scene. This particularity offers the advantage of not requiring periodical operations; in other words, if there is no movement in the scene, the system does not process any data. There are previous approaches for gesture detection using the Dynamic Vision Sensor (DVS) (the same used in the system presented in this manuscript), such as [18], where is presented an event-based gesture recognition system, which processes the event stream using a natively event-based processor from International Business Machines called TrueNorth, they use a temporal filter cascade to create spatio-temporal frames that are the input data to a CNN (Convolutional Neural Network) implemented in the event-based processor, and they reported an accuracy of 96.46%. In [19], a method is proposed to recognize the three hand poses in the game of Rock-Paper-Scissors, and the DVS128 sensor is used to capture frames. For the classification, they use a Bayesian network reporting an accuracy of 89%. Jun Haeng Lee et al. [20] proposed a gesture classification method with two DVSs to obtain a stereo-vision system, they use spike neurons to process the incoming events and an HMM (Hidden Markov Model) based classification stage, and they distinguish between 11 different gestures with movement.

Taking advantage of the non-periodical behavior of the DVS, it is viable to increase parallelism in the algorithms improving the system speed when implemented in FPGA. The novel signature algorithm presented here is more suitable for implementation in digital hardware and does not require a full scanning of the image like the algorithms used in [16], increasing its importance. For the classification of the signs, a feedforward neural network was designed, which has resulted in a very good method for solving pattern recognition problems, yielding very good results. Additionally, this Artificial Neural Network (ANN) is easily implemented in digital hardware because it is mainly composed of simple operations such as addition and multiplication.

Therefore, in this work, we propose an ASL alphabet recognition system implemented in an Altera DE2-115 board that counts with the USB host port required for the communication with the DVS and a proper video deployment port. It is used only one event-based neuromorphic camera. The proposed system does not require any special gloves, color specific background or a desktop computer. Moreover, it includes a novel algorithm to obtain the signatures using the contour. This algorithm is designed for applications in ASL alphabet classification tasks, and the ANN, which is fully implemented in the FPGA. This makes our system suitable for embedded applications, which results in a major portability, including high speed processing with low computational cost implementing customized hardware. As an overall result, this system could be focused as a smartphone accessory.

The remainder of this paper is organized as follows: Section 2 contains a brief explanation of the system presented in this work and the requirements to replicate the experiments. The neuromorphic camera behavior is presented in Section 3. All of the image processing algorithms applied in this work are fully explained in Section 4. The classification system, including the hardware required to implement all of the ANN, is presented in Section 5, followed by the synergy between the hardware accelerators and the processor designed in the FPGA in Section 6. In Section 7, the results obtained in the simulations of the proposed algorithms for every of the 24 ASL signs and the response of the experiments performed with the overall system in a confusion matrix are presented. Section 8 contains the conclusions of the obtained results as well as a comparison with previously proposed hand posture recognition systems, including the future work and its relevance.

## 2. Experimental Methods

**Figure 1:**
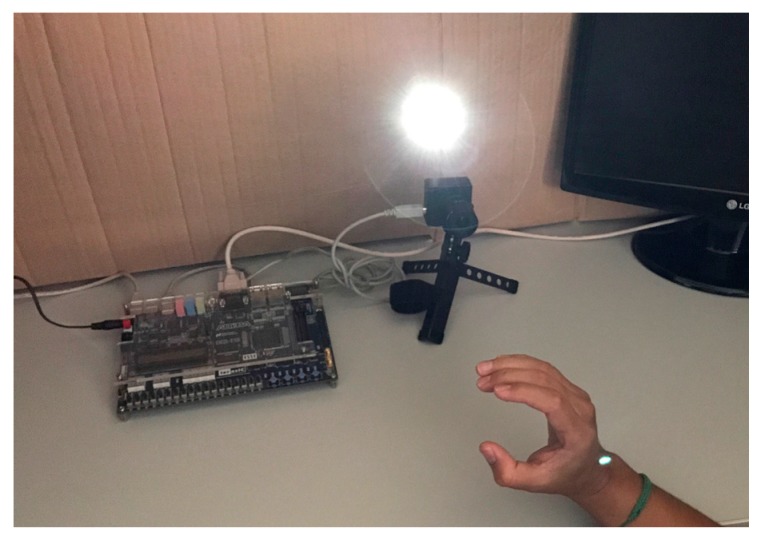
Experimental setup.

The system presented in this paper is fully implemented in a FPGA. It classifies 24 of the ASL alphabet signs using the external contour of the hand in images received from a neuromorphic sensor. It consists of three main sub-systems, the first dedicated to communicating with the camera sensor and receiving the events, the second is the image processing algorithms, including noise filtering and edge sorting, and the last step is the classification using a system composed of a dynamic neurons, static ANN, data adjusting algorithms, and a comparator. As can be seen in Figure 1, the experiments have been performed in a controlled environment, with the sensor placed at approximately 30 cm from the hand user. Since the neuromorphic camera detects changes in the scene luminosity, a white light lamp synchronized with the system focused on the user hand has an on–off controller to ensure the light changes in the hand. This guarantees that all of the pixels of the hand will exceed their internal threshold sending the event.

## 3. Neuromorphic Sensor

The neuromorphic sensor, also called DVS [21], is a sensorial system that consists of a silicon retina formed by pixels in a matrix. The DVS used in this research has a size of 128 × 128 independent pixels. This matrix array is a sensorial retina and every pixel contains asynchronous neuromorphic circuitry. These pixels are sensitive to dynamic luminosity changes in the scene presented to the DVS; in other words, their neuromorphic circuitry allows them to not send data until an event occurs (changes in the scene). Such behavior gives the sensor the peculiarity of very low power consumption, since the data traffic only exists when an event occurs. Therefore, the DVS sends little redundant information in contrast to the conventional digital cameras, which periodically transmit images in frames. Each event carries information, such as the event polarity, the coordinates in the matrix, and a timestamp value.

The event polarity indicates the type of transition occurring in the pixel, where a dark to light transition is called a positive event (ON), and a light to dark is a negative event (OFF). Every event is represented by 32 bits, which are arranged in four bytes. These bytes contain information about pixel position, event polarity and timestamp.

### Neuromorphic Sensor Communication System

The neuromorphic sensor used in this research can be controlled through USB protocol [22], although the DVS developers only provide Windows and Linux drivers; thus, part of this research was to migrate the driver into C code for a processor implemented in the FPGA. In order to communicate with the DVS and manage the whole system, a softcore processor was implemented with a SDRAM (Synchronous Dynamic Random Access Memory) as data and instructions memory that shares a 100 Mhz oscillator with the softcore microprocessor.

**Figure 2:**
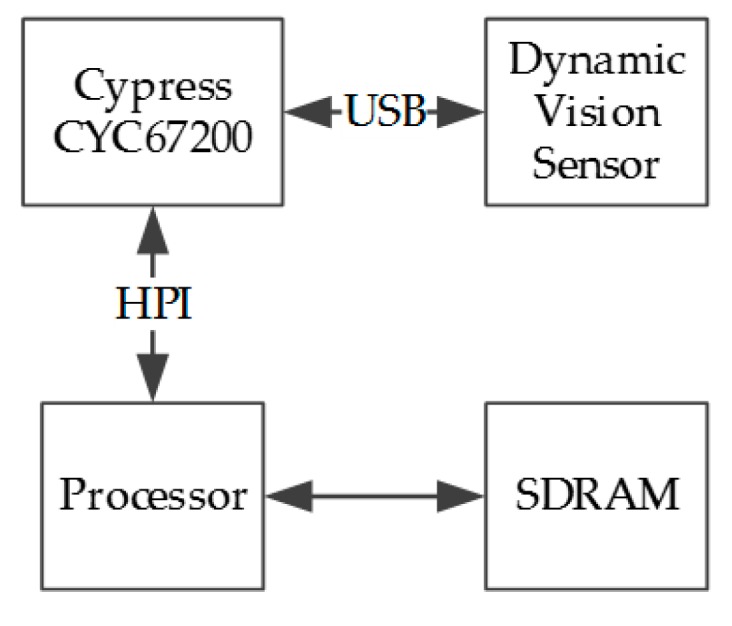
Events reception system block diagram.

The sensorial sub-system consists of a processor with its SDRAM embedded into the FPGA platform used to implement the system. The On-The-Go USB controller generates the differential signals and some communication stages imposed by the USB protocol and the neuromorphic sensor, as can be seen in Figure 2.

## 4. Image Generation and Processing

All of the information sent by the DVS consists of a set of basic events generated by the retina chip as noted above; therefore, the system needs to decode every event as well as create and process the image. When there is only background noise, the period between incoming events is longer because few dynamic luminosity changes are detected by the silicon retina. In contrast, when there is movement in the hand, many events are generated by the retina, representing a major increase in the USB bus traffic, reducing the period between events. When this happens, the system will store and count all of the incoming events within a period of 50 ms, which represents a theoretical rate of 20 fps. Then, it creates the image and processes it.

### 4.1. Noise Filter

**Figure 3:**
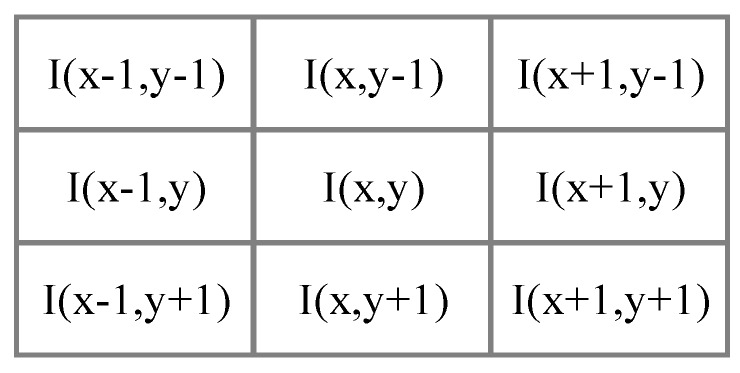
Pixel noise filter neighborhood.

The first step in the image processing stage is to reduce the noise as much as possible in order to decrease useless information, and every image received by the system is submitted to a filter. This process eliminates pixels with few neighbors around the image. These events are usually unwanted noise, and this filter is performed using the Equation (1) (See Figure 3):

(1) $$\bar{I}(x,y)=1\;\mathrm{if}\;\Sigma \;\Sigma \;I(x+i,\;y+j)\geq 5,\;i=j=\{-1,0,1\}.$$

**Figure 4:**
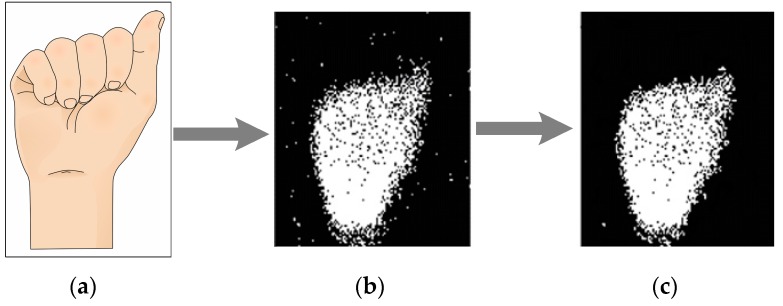
Image to event transition and events filtering of “A” sign, (**a**) ASL alphabet sign； (**b**) raw data sent by the neuromorphic sensor； (**c**) image after filtering.

The resulting image in Figure 4c has very few noisy events and its shape is clear; therefore, it must be tracked and every contour pixel sorted.

This filter eliminates few events, but it is useful for decreasing the time required to execute the next algorithm for edge tracing.

### 4.2. Contour Detection

**Figure 5:**
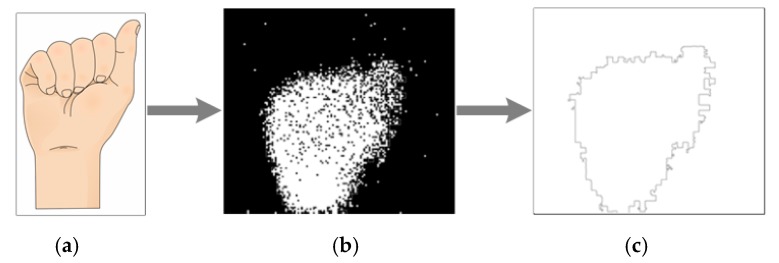
“A” ASL alphabet sign process: (**a**) ASL alphabet sign； (**b**) Dynamic Vision Sensor events； (**c**) resulting image.

The second image processing stage consists of an edge tracing algorithm, used to decrease redundant information. In this step, most of the irrelevant events are eliminated. Figure 5 shows that the only information preserved is the ASL sign external shape. Prior to this stage, an ASL sign is represented by approximately 7000 events, but, after this, it is represented by approximately 500 pixels.

**Figure 6:**
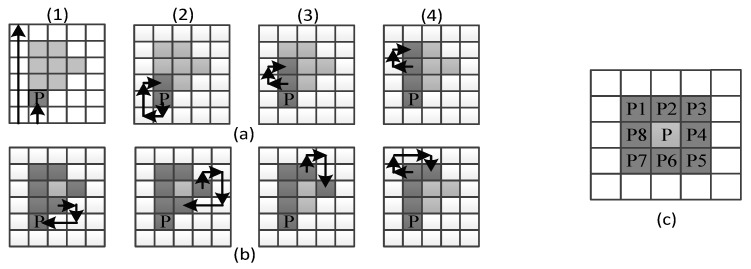
Moore–Neighbor (MN) algorithm demonstration, (**a**) starting steps for MN algorithm； (**b**) last steps for MN algorithm including stopping criterion; (**c**) Moore neighborhood of a pixel P.

The process used to obtain the external shape of every image received by the system is called the Moore–Neighbor algorithm [23]. The idea behind its process is simple, but the implementation is complex. An important term for the understanding of this algorithm is the Moore Neighborhood of a pixel, consisting of the eight pixels that share a side or corner with the pixel, which are called P1, P2, P3, P4, P5, P6, P7, and P8, as can be seen in Figure 6c.

The first step of this algorithm is to find the Start Pixel P; it can be found by checking every pixel from any image corner in any constant direction until the pointer reaches the first object pixel shown in the first step of Figure 6a1.

Once the Start Pixel is found, a set of steps must be performed. Depending on the Moore–Neighbor from which the last contour pixel was accessed, the pointer must check every Moore–Neighbor in a constant direction (clockwise or anticlockwise). For example, for the step (a1) in Figure 6, the Start Pixel was accessed from its Moore–Neighbor P6; thus, the Moore–Neighbors P7, P8, P1, P2, P3, P4, and P5 must be checked in that order until the next object pixel is found. Then, the process repeats until a stopping criterion is found.

The stopping criterion of this algorithm is when the Start Pixel is reached for the second time, as can be seen in the last step of Figure 6b1, or when the only object pixel is that from which it was accessed. This last stopping criterion represents an open contour.

The resulting image is represented by very few pixels, as can be seen in Figure 5c. This represents a huge reduction in the digital hardware required to implement the ANN to carry out the ASL alphabet classification, but is not enough to design an efficient classification system.

There is still redundant information that must be eliminated to properly perform the classification through the ANN.

### 4.3. Characteristic Pixel Extraction

In order to design an efficient system with low computational cost, the quantity of events representing each ASL sign must be decreased without degrading the shape of the object and its characteristics. Therefore, a set of processing stages was developed to reduce the number of pixels representing every contour, which will be called characteristic pixels. With the purpose of reducing characteristic pixels of every shape, an elimination criterion that allows deleting irrelevant information without affecting the contour features must be selected.

**Figure 7:**
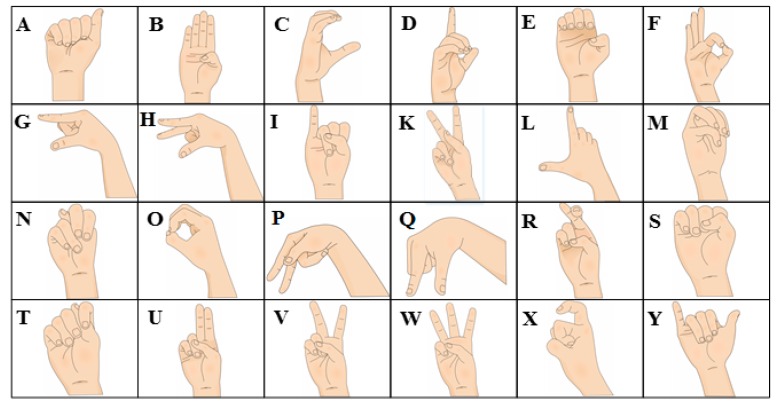
American Sign Language alphabet community used, including letters from A to Y excluding letter J.

The final characteristic pixels quantity directly affects the digital hardware complexity implemented in the classification stage. Thus, some experiments were performed in order to find the minimum pixel quantity while maintaining the features of all the ASL alphabet signs. The first step is to find the elimination criterion. Therefore, it is necessary to detect a pattern that appears in all ASL alphabet signs. Most of the sign shapes have vertical features, for example, the arm direction, finger contours, angles, and the distance between them, as can be seen in Figure 7.

This is the reason for selecting the vertical derivate as the first evaluation criterion, by calculating the vertical slope of the entire contour, all of the vertical vertex pixels of any shape can be found.

The contour slope is calculated using Equation (2), where P represents the characteristic pixels array and n is the size of the characteristic array:

Slope = (P_i+1_(y) – P_i_(y))/(P_i+1_(x) – P_i_(x)), 1 ≤ i < n. (2)

**Figure 8:**
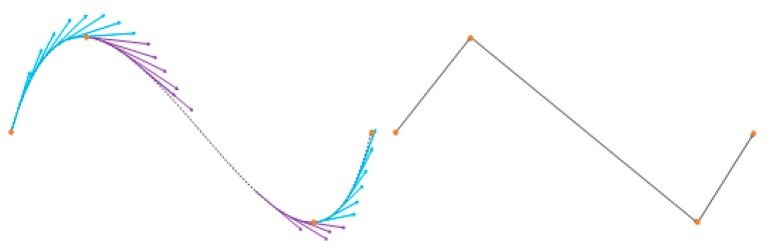
Vertical slope of a curved line.

In Figure 8, where violet and blue arrows represent negative and positive slopes, respectively, the main vertices are characterized for being found in vertical slope sign changes; thus, by storing these points, the quantity of characteristic pixels is reduced without losing the main shape of the line. With this elimination criterion, the quantity of characteristic pixels is reduced to approximately 15. Using this compact characteristic array of pixels, it is feasible to classify the ASL signs.

**Figure 9:**
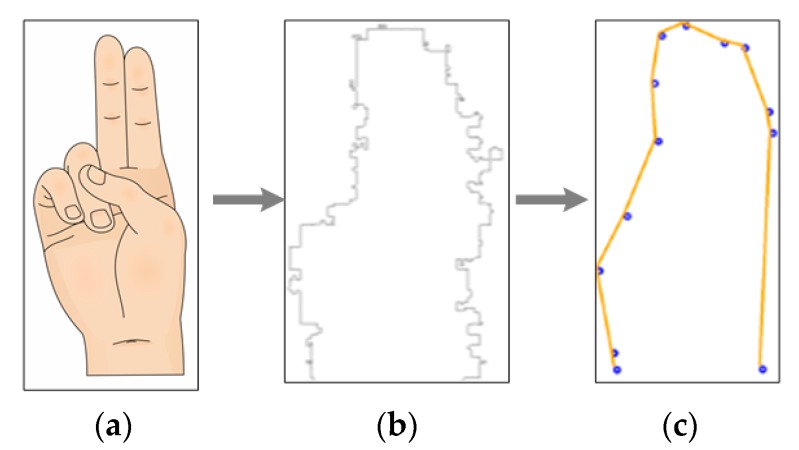
Shape reduction result using the vertical slope algorithm, (**a**) American Sign Language alphabet sign； (**b**) output image of edge tracing algorithm； (**c**) data representation after vertical slope algorithm.

Despite this elimination methodology being effective in reducing unnecessary information, it does not guarantee the quantity of characteristic pixels, obtaining between 5 and 30 pixels in the experiments, 13 in the case of a “U” sign, shown in Figure 9. Since this data is the input for the classification system, it is necessary that every characteristic pixel array be of the same size, a requirement for this ANN.

### 4.4. Characteristic Array Adjusting

In order to select the best size of the characteristic array, a series of experiments was performed with different characteristic pixel quantities, using between 10 and 20 characteristic points. The size selection criterion is the minimum quantity of points necessary to maintain the main features of all the ASL alphabet signs. The artificial neural network will be implemented in digital hardware in the next stage; thus, a larger characteristic array needs a larger ANN, requiring more hardware for its implementation, and, as a consequence, more power will be consumed; therefore, redundant information is particularly harmful to this system.

**Figure 10:**
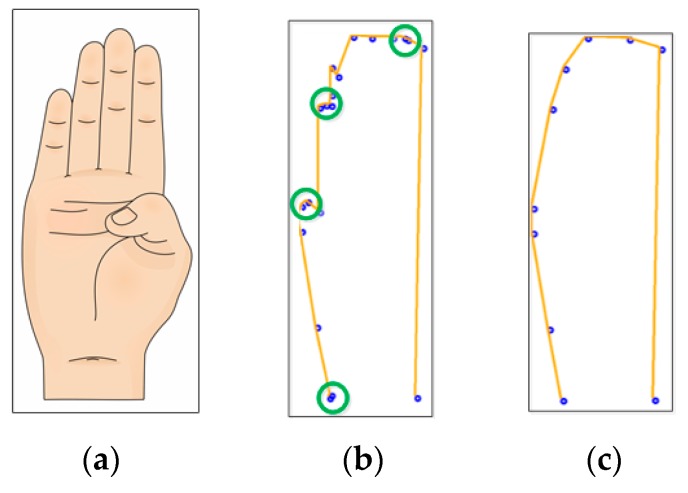
Size selection: (**a**) “B” ASL alphabet sign; (**b**) image processing result with 20 characteristic pixels; (**c**) image processing result with 10 characteristic pixels.

As can be seen in Figure 10b, using a size of 20, regions with a high density of characteristic pixels are created (surrounded in green), representing redundant information, as only one of those pixels gives the information that this point is part of the main shape. The hardware resources required for 20 characteristic pixels do not fit in our FPGA with our ANN architecture. Furthermore, after the vertical slope algorithm, most of the ASL symbols’ characteristic pixels (CP) array contains no more than 20 CP; therefore, more time would be required to add pixels in order to fulfill the size requirement (e.g., 20 to 50). In the other hand, using 10 characteristic pixels, the main shape of all the signs is maintained and well distributed, with very few crowded areas observed in some signs, and so this is the selected size. As noted above, the resulting size of the characteristic array is not guaranteed; nevertheless, only three possible cases may occur: the quantity of characteristic pixels is greater, less or equal to 10.

In the first case, less representative pixels should be eliminated, as they belong to high-density areas. For this, an algorithm calculates the distance between all subsequent pixels, and then one of the two closest characteristic points is eliminated. This is repeated until a size of 10 is reached.

**Figure 11:**
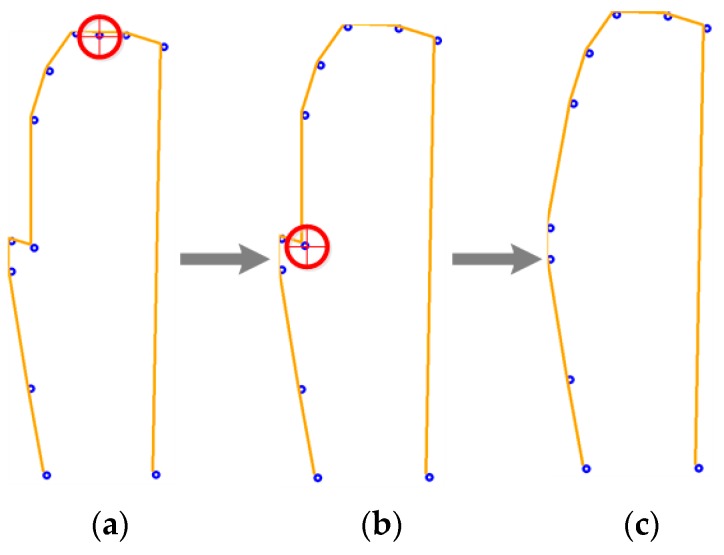
Characteristic array down adjusting, (**a**) characteristic array of size 12； (**b**) characteristic array of size 11； (**c**) final characteristic array of size 10.

Figure 11 graphically represents the resulting array in each cycle of the previously mentioned reducing algorithm. The eliminated points are surrounded by red, the shape on the left represents the result of the vertical slope algorithm with 12 characteristics pixels, and the shape on the right Figure 11c represents the input for the ANN.

**Figure 12:**
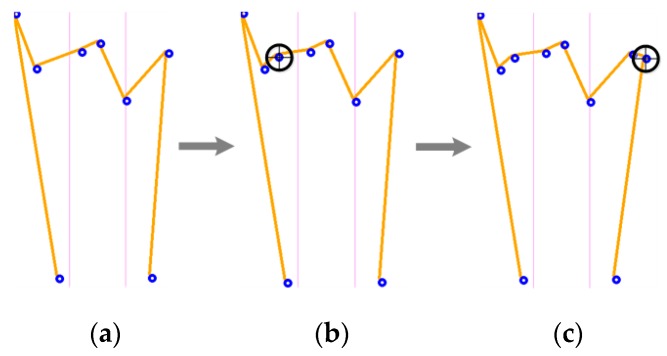
Characteristic array adjustment, (**a**) characteristic array of size 8； (**b**) characteristic array of size 9； (**c**) final characteristic array of size 10.

For the second case, where the array size is smaller than 10, pixels must be added in order to fulfill the requirements of the size imposed by the ANN. For this case, the image is separated into a finite number of sections, and the frontiers between those sections are marked with a violet vertical lines (See Figure 12). Although the quantity of sections can be changed, the more sections there are, the more operations are needed.

Figure 12 represents the Y sign of ASL. It was sectioned into three parts, with the purpose of recognizing which part has a lower quantity of characteristic pixels. Then, pixels are added to this section. Added pixels are selected from the obtained array in the contour detection algorithm explained in Section 4.2, and the only requirement is that the added pixel belongs to the section with fewer characteristic pixels. This allows the system to drastically reduce the probability of creating high characteristic pixels density areas. During the development of this algorithm, it was discovered that some main features lost in the vertical slope algorithm were restored in this pixel addition process.

**Figure 13:**
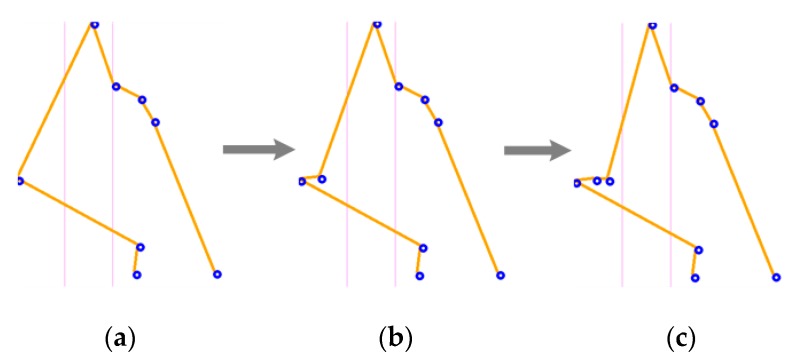
Feature restoration, (**a**) characteristic array of size 8； (**b**) characteristic array of size 9； (**c**) final characteristic array of size 10.

Figure 13 shows the process used to adjust the characteristic array size of the L sign to 10, adding pixels to the section with fewer points. As noted above, the main feature of the L sign is also restored during this process, increasing the importance of this pixel addition criterion.

**Remark** **1.** This algorithm can be used to extract the signature of any object with vertical features, e.g., the ASL alphabet symbols, and standing people, requiring previously a proper segmentation stage.

## 5. Classification System

**Figure 14:**
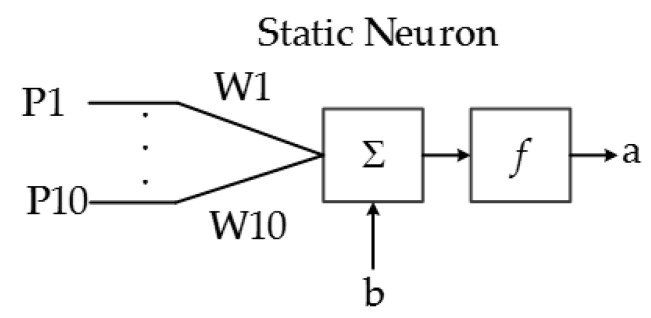
Implemented static neuron architecture.

The main purpose of this system is to classify the ASL alphabet based on the characteristic array of the image; thus, a pattern recognition network is designed in hardware and implemented in a FPGA. ANNs consist of a finite number of basic artificial neurons that exchange data. The static neuron is the most basic block of the ANN implemented in this system, and its structure is presented in Figure 14. The output function f depends primarily on the application of the ANN and the connections of the neuron.

The output equation of a static neuron is:

a = f[Σ(W_k_ × P_k_) + b]. (3)

**Figure 15:**
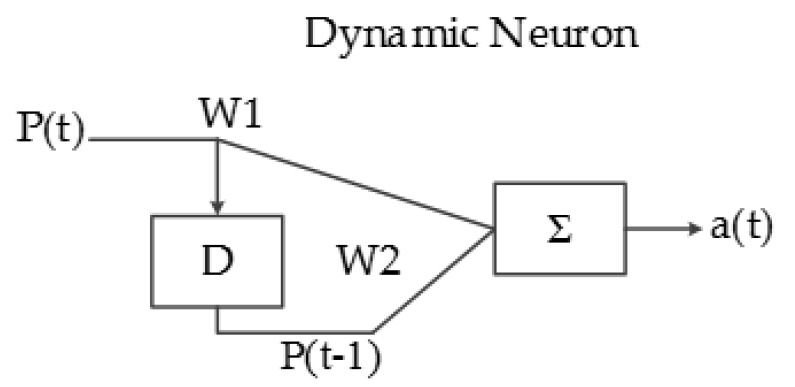
Implemented dynamic neuron architecture.

Another type of artificial neuron implemented in this system is called the dynamic neuron (DN), which has a delayed behavior and the capacity of storing information; the input of the DN is sequential, as can be seen in Figure 15.

The output equation of a dynamic neuron is:

a(t) = W_1_ × P(t) + W_2_ × P(t − 1). (4)

### 5.1. Classification System Specification

In order to reduce the size of the static neural network and digital ports of the main processor, a dynamic neuron was implemented preceding the static ANN. Note that the input for the dynamic neuron must be sequential (property that allows to control the dynamic neuron using only one 32-bit data bus).

**Figure 16:**
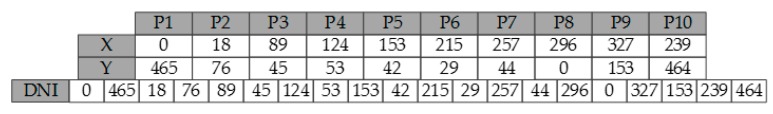
Dynamic neuron input arrangement.

In Figure 16, an example is presented where the first row represents the number of characteristic pixels, the second and third rows represent the *X*- and *Y*-coordinate of each pixel, respectively, and the last row DNI (Dynamic Neuron Input) represents the input data for the dynamic neuron.

Table 1 shows a time representation of the process performed by the dynamic neuron, where valid data ticks represent the product of a valid *X*–*Y* coordinate of each of the 10 characteristic points.

The resulting characteristic array is presented in Table 1. As can be seen, the quantity of data was reduced to half, and, consequently, the hardware necessary to implement the static pattern recognition ANN will be drastically lower.

**Figure 17:**
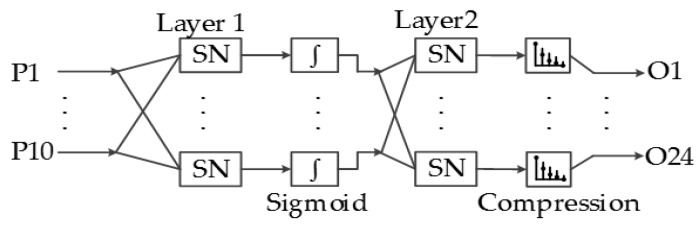
Implemented Artificial Neural Network architecture.

The implemented pattern recognition ANN consists of two layers with 10 and 24 neurons. Figure 17 shows the structure of the ANN implemented in the system presented in this paper, and all of the outputs of the first layer are connected to all of the 24 neurons of the output layer.

The second layer is the output connection of the ANN, which consists of 24 static neurons, where each neuron output represents the similarity of the input to each of the 24 ASL alphabet signs.

### 5.2. ANN Implementation in Digital Hardware

Usually, the image processing algorithms do not require a very high resolution in the data; thus, floating-point representation is avoided in order to reduce hardware requirements. This is why fixed-point representation is commonly used in image processing FPGA implementations; it allows carrying out many fixed-point arithmetic operations with relatively little hardware. The word length used is 32 bits, where most significant bit is the sign bit and the 16 least significant bits are fraction bits. The bits of the integer section will be decreased because this system does not require big values.

**Figure 18:**
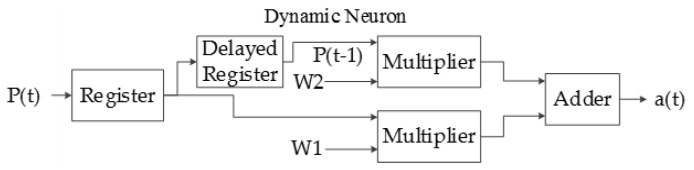
Dynamic neuron hardware.

The dynamic neuron consists mainly of two 32 bit registers connected in series in order to obtain the delayed input as can be seen in Figure 18.

**Figure 19:**
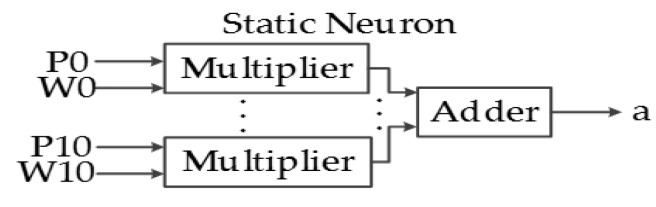
Static neuron hardware.

The logic behind static neurons is simpler because of its combinational behavior, but the hardware required to implement it is substantially larger. Figure 19 shows the block diagram of a static neuron, composed of combinational hardware; some tests will be performed to analyze the consequences of reducing the parallelism in these neurons.

### 5.3. ANN Training

The training of the ANN was performed using a desktop computer and the resulting parameters were stored in registers inside the FPGA.

For the training of the pattern recognition ANN, a community of 720 input images was used, 30 for every ASL sign, and all images reduced to 10 characteristic pixels represented by 10 *X*- coordinates and 10 *Y*-coordinates, using the abovementioned algorithms.

## 6. Hardware–Software Interface

**Figure 20:**
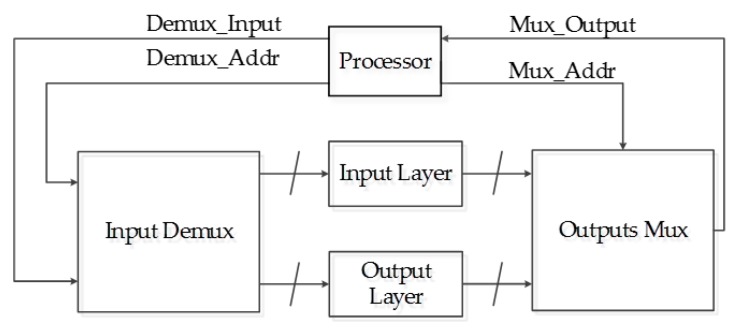
System interconnection.

The processor used to communicate with the DVS is also used to control the ANN implemented in hardware. This represents a reduction in the complexity for the network usage, which also increases the performance of the whole system. The connection between the ANN and the processor is achieved using only four digital buses, two address buses and two data buses. The inputs and the outputs of the ANN are redirected using a demultiplexer and a multiplexer, respectively, as shown in Figure 20.

**Figure 21:**
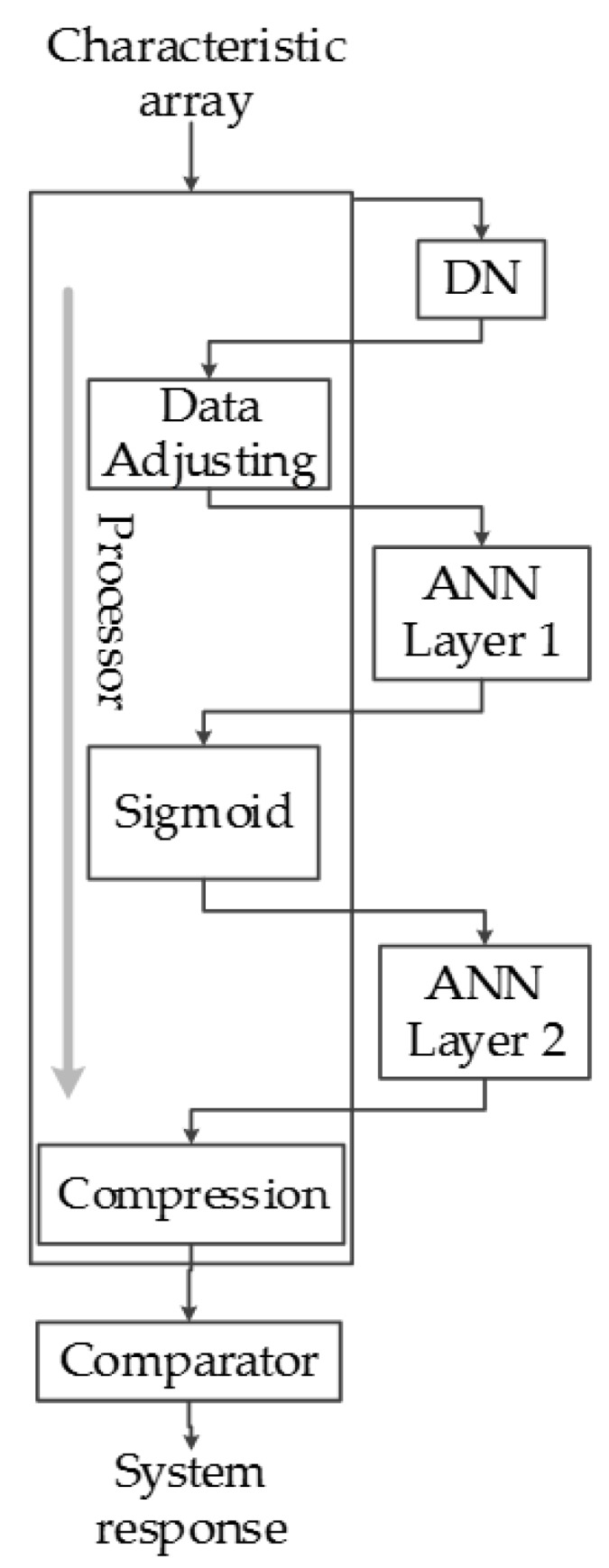
Classification system data flow.

This allows controlling all of the neurons in a relatively simple manner, and the software required to manipulate it is substantially simpler as well. Figure 21 shows the block diagram of the classification system implemented in the FPGA, which contains the software implemented functions including the data adjusting, sigmoid function and data compression (inside the processor block in Figure 21) and hardware blocks (outside the processor block in Figure 21).

## 7. Experimental Results

In order to know the precision of the image processing algorithms, we performed some experiments with a total of 720 images (30 for every ASL sign image) different from the training set under controlled conditions with no background movements and using the method detailed in section two of this paper.

**Figure 22:**
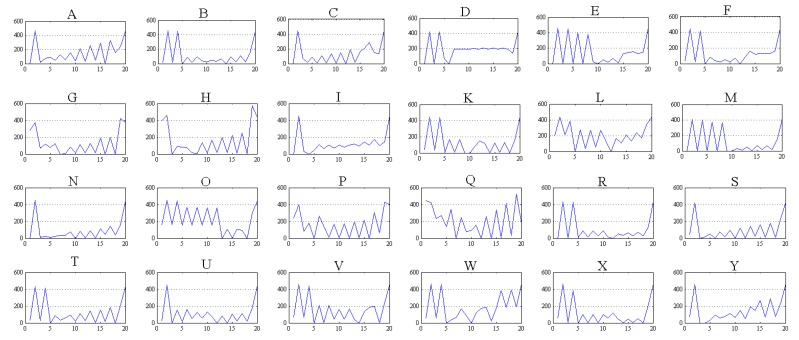
ASL alphabet signs signature used including letters from A to Y excluding letter J, where the *X*-axis represents the 20 *X*–*Y* coordinates of the 10 characteristic pixels in the format (X1, Y1, X2, Y2, …, X10, Y10) and the *Y*-axis represent the value of the coordinates.

Figure 22 shows graphics with the signatures obtained in the image processing stage for the ASL alphabet signs, whose vectors are the input for the classification system. As shown in Figure 22, the letters “Q” and “L” have few similarities with other ASL sign arrays.

These graphs represent the results obtained in simulations of the 20 *X*–*Y* pair coordinates for a single ASL alphabet sign.

In the confusion matrix (Table 2), where columns represents target and rows represent system response, we can see that the best cases are the letters “L”, “Q”, and “T”, mainly because these signs have very few similarities with the others and their vertical features. Conversely, the worst case is the letter “B”, with only a 30% accuracy.

The overall accuracy percentage is around 80%, which is an acceptable result for the simplicity and easy implementation of the algorithms and the low resolution of the available neuromorphic sensor. The results obtained of the whole classification system are very dependent on the sign presented. The letter “B” was misclassified with many different signs, which we attribute to two factors: the low resolution of the DVS and the need for more robust characteristic pixel classification algorithms. Nevertheless, this is the first work that uses a Dynamic Vision Sensor with the proposed algorithms for the classification task, while proving that good results can be obtained using a non-frame-based sensor, reducing the need to periodically process images.

Some previously presented systems [8] reported an accuracy around 53% using Microsoft Kinect for depth information. Kumar et al. achieved an accuracy as high as 90.80%, but they use two different sensors and their system was implemented in software requiring a computer, while other presented systems require specially colored gloves [15].

## 8. Conclusions

Hearing loss is a problem that affects 5% of the world population. Many efforts have been made to improve both the accuracy and the performance of the hand posture recognition tasks, but almost all of them require computers with operating system, making them unsuitable for embedded applications, which represents a more realistic approach because of the mobility of those platforms. For this reason, this work focuses on the development of a system in a FPGA, which can be implemented in an embedded platform with very low computational requirements. Additionally, the usage of the DVS with the abovementioned algorithms is a novel approach for ASL alphabet recognition, which significantly reduces redundant processing.

The novel algorithms developed for this system were specially designed for American Sign Language classification using contours, which have proven to be very efficient for obtaining the feature vector for classifying the ASL alphabet using artificial neural networks. These vectors are also very suitable for use as a training set for the ANN, so few neurons are required for its implementation.

The system accuracy is acceptable, considering the few operations required by its algorithms; the process performed to classify every image requires only one full image scan in the noise filtering step, which drastically reduces the operations required and the time taken to process an input image. With respect to RGB image processes such as gray scaling, morphological operations, and kernel based algorithms requiring full scanning and processing of the image, this has repercussions not only on the time needed for its algorithms, but also on the memory, which must be resized according to the input image.

The accuracy of the overall system can be improved when the aforementioned main weaknesses are solved, noise filter stage is not robust enough to ensure a fast edge tracing process, leading to a bigger delayed response mainly because of this reason. Most of the lost symbol features are eliminated in the vertical slope algorithm because they are not vertical features. This results in a weak signature extraction in some ASL symbols, which affects the ANN training performance as well as the overall accuracy. Due to the nature of the signature extraction algorithm, it is not invariant to moderate orientation changes of the ASL symbol. Nevertheless, the images obtained for testing the system presented an angular variation of approximately ±8°. However, if this algorithm is applied to more rotation variant applications, it should be improved to decrease rotation sensitivity.

There are still some tests and modifications to be made in order to increase the accuracy while maintaining the system simplicity. For example, changing the fixed point format could reduce required hardware. Adding more characteristic pixel selection criteria could lead to a more robust events processing stage, so horizontal features can be captured, yielding to a robust algorithm against hand rotations. An algorithm for detecting the time for the initial and final frames in the neuromorphic sensor for real-time application is another issue that remains.

Other ANN architectures are being tested, including Convolutional Neural Network (CNN), and this architecture processes the input data without requiring pre-processing, avoiding in that way the noise filter. The Recurrent Neural Network (RNN), which has a sequential nature, therefore, is more suitable for the asynchronous spikes stream sent by the DVS.

## Figures and Tables

**Table 1:** Dynamic neuron time representation.

Time(t)	P(t)	P(t-1)	a(t)	Valid Data
1	0	0	0	
2	465	0	1658.7	√
3	18	465	1103.5	
4	76	18	311.33	√
5	89	76	487.33	
6	45	89	359.43	√
7	124	45	542.90	
8	53	124	466.19	√
9	153	53	664.23	
10	42	153	491.77	√
11	215	42	860.81	
12	29	215	583.97	√
13	257	29	981.58	
14	44	257	731.34	√
15	296	44	1154.2	
16	0	296	661.55	√
17	327	0	1166.5	
18	153	327	1276.6	√
19	239	153	1194.5	
20	464	239	2189.3	√

**Table 2:** Confusion matrix.

	A	B	C	D	E	F	G	H	I	K	L	M	N	O	P	Q	R	S	T	U	V	W	X	Y
A	25	0	1	0	0	1	0	0	2	0	0	2	0	0	0	0	0	0	0	0	0	0	0	0
B	0	9	0	3	0	0	1	1	0	0	0	3	0	2	0	0	0	0	0	3	0	0	0	0
C	0	0	29	0	0	1	0	0	1	0	0	0	0	0	0	0	0	0	0	0	0	0	0	0
D	0	5	0	20	0	0	0	1	0	0	0	0	2	0	0	0	0	1	0	4	0	0	0	0
E	0	0	0	0	22	0	0	0	0	0	0	1	0	2	0	0	0	0	0	1	0	0	0	0
F	0	0	0	1	1	23	0	0	0	0	0	1	0	2	0	0	0	0	0	0	0	0	0	1
G	0	0	0	0	0	0	27	1	0	0	0	0	0	1	0	0	0	0	0	0	0	0	0	0
H	0	0	0	0	0	0	1	24	0	0	0	0	0	0	0	0	0	0	0	2	0	0	0	0
I	0	1	0	0	0	0	0	0	22	0	0	0	3	0	0	0	1	0	0	0	0	0	0	0
K	0	0	0	0	0	0	0	0	0	27	0	0	0	0	1	0	0	0	0	0	7	0	0	0
L	0	0	0	0	0	0	0	0	0	0	30	0	0	0	0	0	0	0	0	0	0	0	0	0
M	1	7	0	1	4	2	0	0	1	0	0	23	0	3	0	0	0	1	0	0	0	0	0	1
N	0	0	0	0	1	0	0	0	1	0	0	0	17	0	0	0	0	0	0	1	0	0	4	0
O	1	4	0	0	2	3	0	0	1	0	0	0	0	19	0	0	0	0	0	0	0	1	0	0
P	0	0	0	0	0	0	0	0	0	0	0	0	0	0	29	0	0	0	0	0	0	0	0	0
Q	0	0	0	0	0	0	1	3	0	0	0	0	0	0	0	30	0	0	0	0	0	0	0	0
R	0	1	0	1	0	0	0	0	0	0	0	0	0	0	0	0	26	0	0	3	0	0	4	0
S	2	0	0	0	0	0	0	0	0	0	0	0	0	1	0	0	0	27	0	0	0	0	0	0
T	0	0	0	0	0	0	0	0	0	0	0	0	6	0	0	0	0	0	30	0	0	0	0	0
U	0	3	0	4	0	0	0	0	0	0	0	0	0	0	0	0	2	0	0	16	0	0	1	0
V	0	0	0	0	0	0	0	0	1	3	0	0	0	0	0	0	0	0	0	0	22	1	0	0
W	0	0	0	0	0	0	0	0	0	0	0	0	0	0	0	0	0	0	0	0	1	28	1	0
X	0	0	0	0	0	0	0	0	0	0	0	0	2	0	0	0	1	0	0	0	0	0	20	0
Y	1	0	0	0	0	0	0	0	1	0	0	0	0	0	0	0	0	1	0	0	0	0	0	28
